# Fast and Accurate Construction of Ultra-Dense Consensus Genetic Maps Using Evolution Strategy Optimization

**DOI:** 10.1371/journal.pone.0122485

**Published:** 2015-04-13

**Authors:** David Mester, Yefim Ronin, Patrick Schnable, Srinivas Aluru, Abraham Korol

**Affiliations:** 1 Institute of Evolution, University of Haifa, Haifa, Israel; 2 Center for Plant Genomics, Iowa State University, Ames, Iowa, United States of America; 3 Department of Electrical and Computer Engineering, Iowa State University, Ames, Iowa, United State of America; Ben-Gurion University, ISRAEL

## Abstract

Our aim was to develop a fast and accurate algorithm for constructing consensus genetic maps for chip-based SNP genotyping data with a high proportion of shared markers between mapping populations. Chip-based genotyping of SNP markers allows producing high-density genetic maps with a relatively standardized set of marker loci for different mapping populations. The availability of a standard high-throughput mapping platform simplifies consensus analysis by ignoring unique markers at the stage of consensus mapping thereby reducing mathematical complicity of the problem and in turn analyzing bigger size mapping data using global optimization criteria instead of local ones. Our three-phase analytical scheme includes automatic selection of ~100-300 of the most informative (resolvable by recombination) markers per linkage group, building a stable skeletal marker order for each data set and its verification using jackknife re-sampling, and consensus mapping analysis based on global optimization criterion. A novel Evolution Strategy optimization algorithm with a global optimization criterion presented in this paper is able to generate high quality, ultra-dense consensus maps, with many thousands of markers per genome. This algorithm utilizes "potentially good orders" in the initial solution and in the new mutation procedures that generate trial solutions, enabling to obtain a consensus order in reasonable time. The developed algorithm, tested on a wide range of simulated data and real world data (*Arabidopsis*), outperformed two tested state-of-the-art algorithms by mapping accuracy and computation time.

## Introduction

Numerous projects have generated an abundance of genetic mapping data. Consequently, within a given species, many multilocus maps have been constructed. The quality of these maps varies broadly among populations, marker sets, and the mapping software that was used for map generation. This complexity has led to inconsistencies among different versions of genetic maps for the same organism. The maps created by different research groups for an organism could contain different numbers of markers, and not all markers may be present in each map. Markers scored for two or more mapping populations can be referred to as *shared*, in contrast to *unique* markers that are scored in only one population. Consequently, a consensus map is constructed for shared markers may include many more markers than each individual map. Because of errors in the individual maps, the orders of certain shared groups of markers may differ among the mapping populations (we call them *conflicting markers*).

Consensus genetic maps, by definition should not have conflicted orders for shared markers. In dealing with this problem, the order of unique markers should also be optimized accordingly. Modern mapping projects can include tens of thousands of markers and more. The problem of consensus genetic mapping is by far more challenging than genetic mapping based on one data set which is also not simple. Mathematical complexity of consensus genetic mapping led to the use of different local optimization algorithms for conflicted marker regions (with tens of markers only) along the chromosome and resolving each conflict separately via heuristic and exact methods. Such an approach implies that resolving a conflict in a certain chromosomal region will not affect the order of markers outside the region; this assumption may not necessarily be true. Another way to solve the problem is reducing the consensus-mapping problem to single-population ordering via constructing a synthetic distance matrix from all datasets, hence avoiding situations with conflicting markers. These two ways to solve consensus genetic mapping are present in [1–16]. Several software packages implementing these approaches are known, e.g., JoinMap (http://www.kyazma.nl/); CarthaGene (http://www.inra.fr/internet/Departements/MIA/T//CarthaGene/) and MultiPoint (http://www.MultiQTL.com). This problem can also be addressed by using some solver programs, e.g., MergeMap (http://mergemap.org/); ILPMap [13], and DAGGER [15].

Before starting the consensus mapping analysis for a set of mapping projects, high-quality individual maps should be constructed for each projects. In recent years, new chip-based platforms have become widely available for scoring thousands of single nucleotide polymorphic markers (SNPs) in a wide spectrum of both model and non-model organisms. This poses new challenges for building high-density genetic maps and integrating mapping data from different labs and mapping populations. To build genetic maps with 10^5^–10^6^ markers per genome new, fast, and accurate algorithms are required. There are a few algorithms that can deal with mapping problems of such sizes within a reasonable time, e.g., MSTmap, Lep-Map, and MultiPointULD. MSTmap is based on using the minimal spanning tree (MST) algorithm [17]. Lep-Map immitates MST construction in finding a feasible initial marker order and then improves the solution by using maximum likelihood analysis and approximate TSP heuristics [18]. Missing and inaccurate distances are refined based on nearby markers in partial solution (similar to multi-point linkage analysis). After the initial order has been established, local changes are applied to maximize the likelihood of the final order [18]. Another approach [19, 20] to build genetic maps with big datasets is used in MultiPointULD software package which was demonstrated by us on PAG XXII conference (see C04 Computer Demos in https://pag.confex.com/pag/xxii/webprogram/Paper9487.html).

New genomic technologies have opened unprecedented possibilities in building ultra-dense genetic maps. Theoretically, in absence of genotyping errors and modest sample sizes, the vast majority of markers in big SNP mapping datasets will remain inseparable by recombination. Real situations are complicated by genotyping errors, which “diversify” a certain part of the markers that would be identical in error-free situations, leading to false recombinants, wrong marker orders and tremendous inflation of map lengths. Bearing this in mind, we suggested a simple approach for selecting error-free markers based on the assumption that the occurrence of such markers should be higher among groups of absolutely linked (co-segregating) markers [19, 20]. The developed algorithm, implemented in MultiPointULD (http://www.MultiQTL.com) software, enables mapping big sets of markers (∼10^5^–10^6^). Unlike some other algorithms for building ultra-dense genetic maps, the proposed approach does not need any prior information (e.g., anchor markers), and hence can be applied to genetically poorly studied organisms. This approach proved efficient in mapping analysis of SNP markers generated using both genotyping-by-sequencing (GBS) or chip-based genotyping platforms [19, 20].

Chip-based analysis for scoring SNP markers allows producing high-density genetic maps with a relatively standardized set of marker loci for different mapping populations. The availability of a standard high throughput mapping platform simplifies the consensus analysis by removing the computation challenges caused by the presence of a considerable proportion of unique (population specific) markers as part of the optimization problem, which would be inevitable in cases of insufficient density of shared markers. The ever-increasing number of markers available by chip technology allows ignoring unique markers at the stage of consensus mapping thereby reducing mathematical complicity of the problem and in turn analyzing bigger size mapping data using global optimization criteria instead of local ones.

In this paper, we introduce a new effective algorithm for consensus genetic mapping with a global optimization criterion, which is especially suitable for SNP datasets with a negligible proportion of unique markers. The algorithm was tested on a wide range of simulated datasets and publically available data from 17 F_2_ populations of *Arabidopsis* [21] and proved more efficient compared to two state-of-the-art algorithms [11, 13, 14].

## Materials and Methods

### Consensus genetic mapping for data containing both shared and unique markers

**Fig 1:**
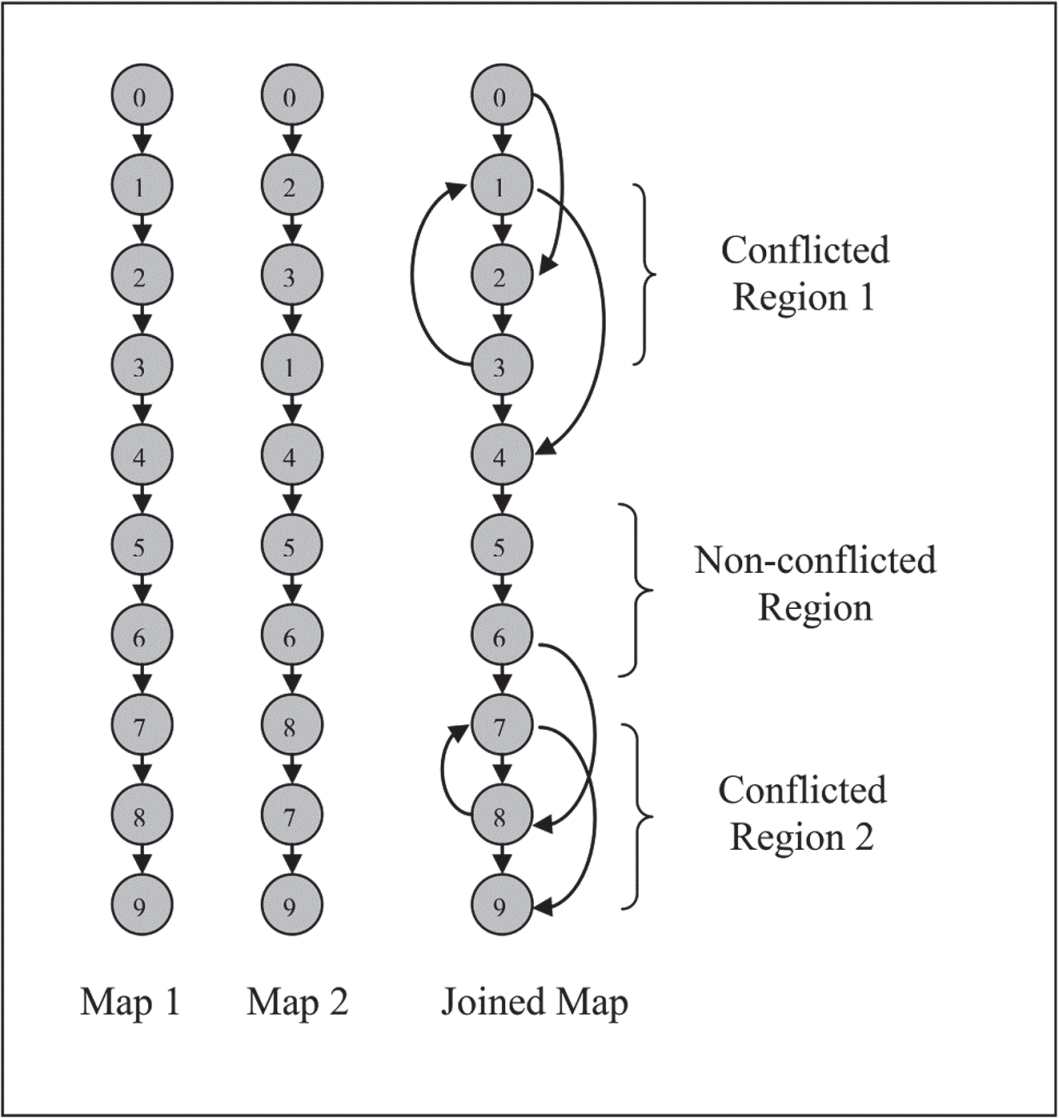
Representing a set of two individual maps as a directed acyclic graph (DAG). Two single maps (map1 and map2) are joined as a DAG. The joint map contains two conflicted regions.

For constructing consensus genetic maps in the presence of unique and shared markers, we can distinguish two major approaches:

- Representing the set of individual maps as a directed acyclic graph (DAG), in which some markers can have conflicted orders. In the illustration of a DAG (Fig 1), the integrated map has two conflicted regions in which shared markers appear in conflicting orders.

There are some ways to resolve conflicting orders via this approach: to remove a minimum weighted set of feedback edges [6], to resolve conflicts by deleting a minimum set of marker occurrences [11, 14], or to minimize breakpoint vertex set [13]. Note that removing even one marker in the integrated map requires re-analysis of the individual maps harboring this marker. One cannot rule out that removing such a marker will not generate new conflicts. The required re-analysis step is absent in the graph-theoretic approach. This approach does not use the whole matrix of marker distances. Instead, the accumulated distance between the markers along the map is used. Depending on the problem size, a heuristic algorithm or an exact solver are employed in the approach.

**Fig 2:**
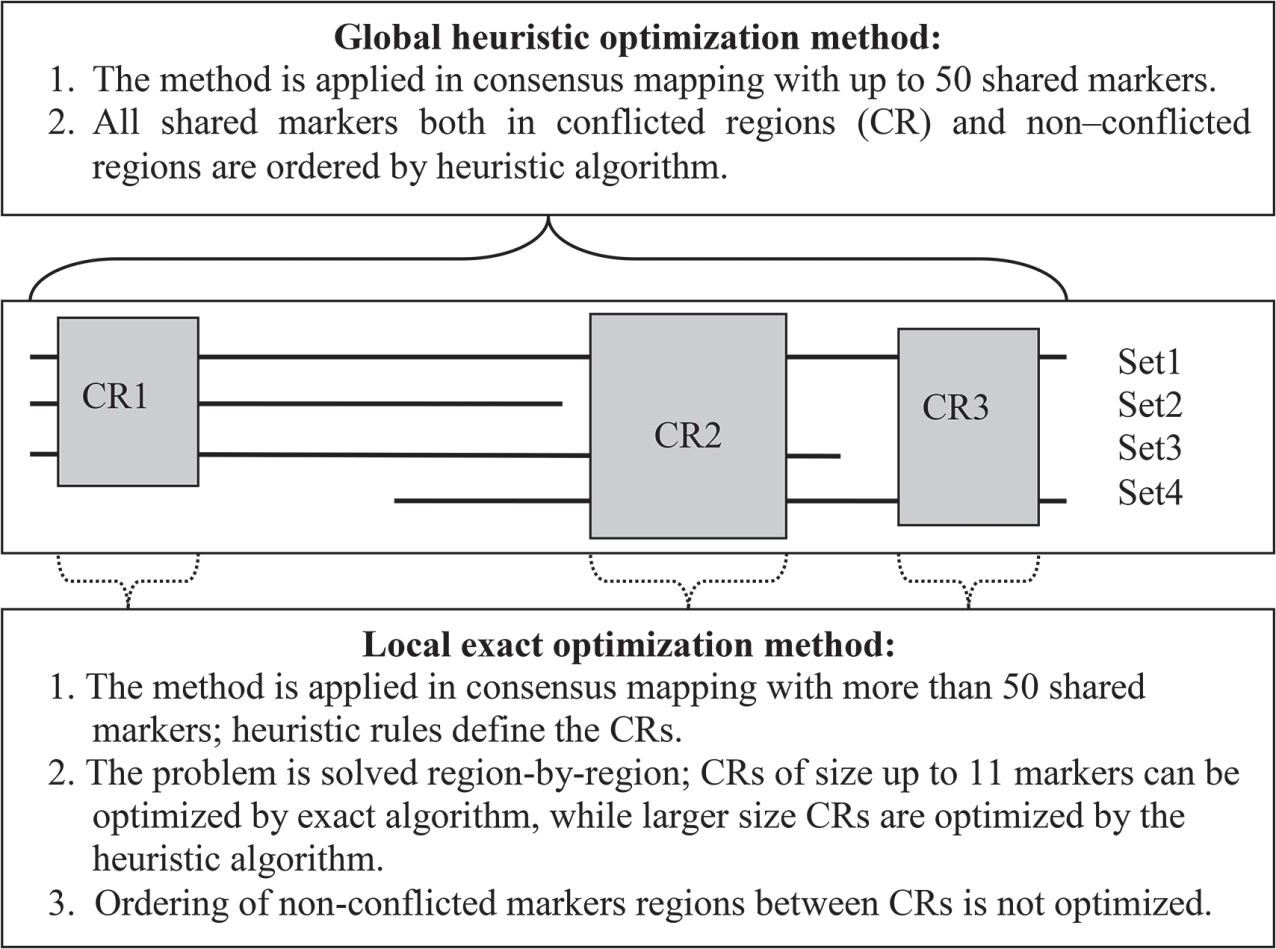
Main features and field of application of the two optimization methods (Globalheuristic and Local exact) for solving multilocus consensus mapping problems.

- Reducing consensus mapping to a specific (constrained) version of the traveler salesperson problem (TSP) that can be referred to as *synchronized TSP*. To solve the problem we searched for the best multilocus order corresponding to the minimum weighted sum of map lengths among non-conflicting orders [8, 9, 12, 16 ]. This approach uses the whole matrix of marker distances for each mapping population included in the analysis. For consensus mapping we employed two methods, *local* and *global*, and two optimization algorithms (exact and heuristic) (Fig 2). The main reason for using the local analysis is the complexity of the optimization problem. In this case, to build the consensus map it is necessary to define the conflicted regions along the chromosome and resolve each conflict separately. This procedure should also include ordering of the unique markers found in the conflicted regions, which significantly complicates the optimization process and the very possibility to use global optimization criteria.

### The proposed scheme for consensus mapping of data without unique markers

The proposed analytical scheme for consensus mapping for chip-based genotyping data includes three phases. Phase 1 is the automatic selection of ~100–300 of the most informative markers per linkage group [19, 20]. In phase 2, a stable skeletal marker order for each data set is constructed and verified using jackknife re-sampling [9, 22–23]. The final phase is solving the consensus-mapping problem based on a novel Evolution Strategy (ES) optimization algorithm with a global optimization criterion.

A mathematical formulation of the problem for phase III is as follows. Let *Q*_*i*_ be the set of all markers of the analyzed chromosome scored for the *i* ^th^ population (dataset). Then, *Q* = ∪(*Q*_*i*_∩*Q*_*j*_) (for all pairs *i* ≠ *j*) is the combined set of markers that appear in at least two populations of the consensus mapping problem. Let denote by *G* the set of all possible orders *g*_*j*_ of shared markers. Then, the optimal order g* of shared markers can be defined as one from the set *G* minimizing the sum

$$S\left(g_{j}\right)=\sum_{i}w_{i}S_{i}\left(g_{j}\right),g_{j}\in G$$ 1

Here *S*_*i*_ is the sum of all recombination rates between the consecutive markers along the map for the *i* ^th^ set (population) of the analyzed chromosome; *w*_*i*_ is the weight of *i* ^th^ set in the optimization criterion that can characterize the quality or reliability of the data [16]. This criterion can be modified to take into account the variation in the quality of original datasets (e.g., population size and/or marker quality). To solve the problem, based on optimization of the global (for the entire chromosome) criterion, we have developed a new ES algorithm described below.

### A new ES algorithm for consensus mapping using a global optimization criterion

ES is a heuristic algorithm mimicking natural population processes. The numerical procedures in such an optimization employs simulation of “mutation processes” as a source of trial solutions (“genotypes”), followed by selection of the fittest genotype based on obtained values of the optimization criterion. For our problem, ES generates possible orders of shared markers *g*_*j*_ ∊ *G* and then selects the best one by the criterion (1). In contrast to the local methods (i, ii), in our analytical scheme, the optimization is applied to the entire set *G* of markers of the considered linkage group, without subdividing it into conflicted and non-conflicted regions. Theoretically, to get the exact solution to the consensus genetic mapping problem with a global criterion, we must generate all |*Q*|!/2 ∊ *G* possible orders of shared markers. Although the DAG contains significantly less possible orders, it can serve as a source of good solutions. In our ES optimization algorithm, the generation of possible solutions *g*_*j*_ is performed on both *G* and *G*_*DAG*_ in parallel. The algorithm includes a new multi-parametric mutation mechanism for the generation of possible solutions *g*_*j*_ from the current best solution *g*_*best*_ on *G* and *G*_*DAG*_ via an operator with four components:

$$g_{j}=\text{M}\{g_{best},\alpha ,\beta ,\gamma \},$$ 2

where *α* component defines the variable neighborhood to be targeted by a mutation mechanism; *β* defines the type of mutation procedure; and *γ* defines the mutation size on the selected neighborhood, i.e., how many components in *g*_*best*_ will change their positions. During mutation step *t* of our new ES algorithm, the three mutation procedures generate a new population of *λ* solutions *g* based on the current *g* ^*best*^. Based on (**1**/***λ***)-selection strategy, the ES algorithm now selects the best solution from ***λ*** generated solutions to use in the next round of optimization steps. More details on our implementation of the Evolution Strategy for solving combinatorial optimization problems are provided in file S1 Text.

In the proposed ES algorithm, an initial solution *g* ^0^ is defined from a combination of marker orders of the set of *n* individual maps with |*Q*| shared markers. In the first step of the algorithm, the *initial solution* algorithm randomly selects an individual map *i* from the set of *n* maps and inserts its marker orders into the new initial solution *g*_*k*_. After excluding map *i* from the set *n*, another individual map *j* is randomly selected from the remaining *n*-1 maps and the order of the *j* ^th^ map markers not yet included into *g*_*k*_ is appended to the end of *g*_*k*_. The process of appending marker orders by using the remaining maps is repeated until all |*Q*| markers will be inserted into *g*_*k*_. Obviously, not all individual maps may have the chance to “delegate” their marker orders to *g*_*k*_ during such a cycle of *g*_*k*_ enrichments. By this reasoning, the algorithm repeats the generation of solutions *n* ^2^ times and the best one of the obtained *g*_*k*_, by criterion (1), is selected as an initial best solution *g* ^0^. The main steps of the ES algorithm are shown below:

1. Define initial solution: *g* ^0^ = best of (*g*_*k*_);

*g* ^best^ = *g* ^0^; *t* = 0; *S*(*g* ^best^) = ∑_*i*_ *w*_*i*_ *S*_*i*_(*g* ^*t*^)

2. *t* = *t*+1

3. Generate new population of size *λ* of individuals *g* ^*t*^ on current *g* ^best^ via muti-parametric mutator M{*g* ^best^, *α*, *β*, *γ*} 

4. For each *g* ^*t*^ in *λ*

{

5. Define total length of the consensus map *S*(*g* ^*t*^)

6. If *S*(*g* ^*t*^)<*S*(*g* ^best^) then *g* ^best^ = *g* ^*t*^

7. Local search on *g* ^best^

}

8. If not finished then go to step 2

As a minor “curing” stage, a fast local search applied on small neighborhood (size 5–10 markers) tries to improve *g* ^best^ via Reinsert [24] and 2-Opt procedures [25]. This local search optimizes the solution between conflicted and non-conflicted regions.

### Mutation mechanism of ES algorithm proposed for consensus genetic mapping

To obtain the exact solution of consensus genetic mapping by the approach (ii), all possible marker orders in the criterion (1) should be tried. One of the ways to get a satisfactory approximate solution of this computationally challenging problem by using heuristic approaches (as the one proposed in our previous publications [9, 16]) in reasonable time is to try only "promising" marker orders. An additional way to accelerate the optimization process during a generation of new trial solutions is to use the marker orders present in the original single maps. These orders comprise a set of "good orders" because the initial maps have already been optimized and none of the potential consensus solutions can be shorter than the sum of the initial lengths. In this paper, we adapted the idea of using the initial orders in two new mutation procedures referred to as *sequential constructing mutation procedure* (SCMP) and *reference-based constructing mutation procedure* (RCMP). Thus, in the new ES algorithm, three types of mutation procedures, RMP (random mutation procedure from [9, 16]), SCMP, and RCMP, create the population list *P* of size *λ* = *λ*_RMP_ *+λ*_SCMP_ *+λ*_RCMP_. RMP generates random combinations of shared markers on the full set of possible marker orders *G* enabling to try marker orders not present in the DAG. Similar to the idea developed earlier [6, 10], SCMP and RCMP generate marker orders by random voting on the restricted area of possible marker orders presented as the list of marker neighbors *L*_*ij*,_. With our current approach, the list *L*_*ij*_ includes direct and reversed arcs of the DAG.

**Fig 3:**
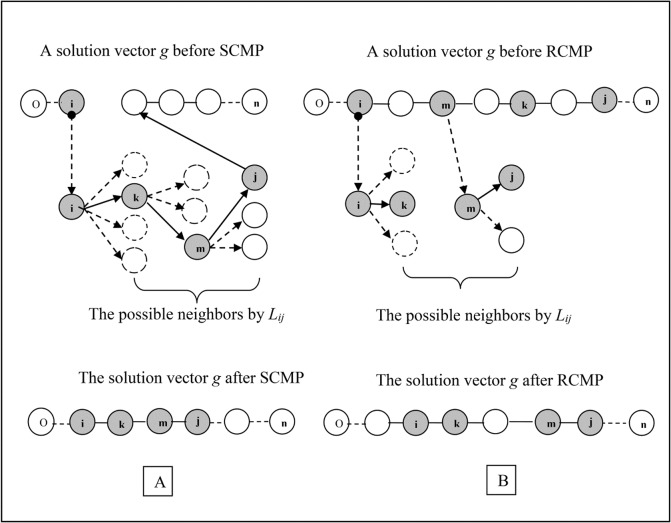
The idea of new mutation procedures based on using the list of neighbors *L_ij_*. (A) Sequential constructing mutation procedure (SCMP): SCMP generates a new random sequence of markers (*i-k-m-j*) from a randomly selected marker (*i*) of the solution vector *g*. (B) Reference-based constructing mutation procedure (RCMP): RCMP reinserts two randomly defined markers (*i*) and (*m*) of g to other positions.

Two different ideas are used in the SCMP and in the RCMP type mutation procedures. The heuristic in the SCMP supposes that the marker at a random position *p*_1_ of the solution vector *g* is placed correctly, while some of the following markers are not correctly ordered. Therefore, SCMP generates and tries other sequences of the markers (by the list of neighbors *L*_*ij*_) beginning from randomly selected position *i* (Fig 3). After each inserting of a marker from the list to the sequence, the new solution is included to a population list *P*. The number of components (markers) in the generated sequence (variable mutation neighborhood) and the size of the generated population may vary from 2 to *n*. The procedure of generating of such a sequence is terminated by exhausting the *L*_*ij*_ list. As one can see in Fig 3, three components (markers *k*, *m*, and *j*) were removed from their positions in vector *g* ^*t*^, and placed consequently after component *i*.

In contrast to SCMP, the idea of RCMP heuristic is based on the assumption that the marker at a random position *p*_1_ of the solution vector *g* is placed incorrectly. RCMP procedure is similar to RMP, but unlike RMP the new random position *p*_2_ is defined according to the list of neighbors *L*_*ij*_ (Fig 3). SCMP and RCMP work on a considerably smaller solution space, thereby allowing significant acceleration of the optimization process. In addition to the known “transitions” utilized in *L*_*ij*_, random transitions leading to a solution not achievable via *L*_*ij*_, can be generated by RMP. All our algorithms were implemented in Visual Basic 6 on Windows 7 desktop PC with 4-core 2.66GHz Intel Pentium processor and 8GB RAM.

### Data sets employed in the study

To test the proposed algorithm and compare it with two state of the art algorithms, we employed simulated datasets and mapping data from 17 F_2_ populations of *Arabidopsis* [21].The simulated datasets included examples with five mapping populations. In Group 1, ten examples with different distribution of recombination rates and interference values along the chromosome were simulated, with 100 markers scored without errors. Similarly, in Group 2, ten examples we simulated with errors in three out of five populations (80% of markers were scored with an error rate of 10%). In addition, we also simulated two examples with five populations with 200 and 500 markers and the same level of genotyping errors. For each marker, 20% of data points were simulated as missed. Two different uniform distributions were used for recombination distances (cM) between neighbor markers: for Group 1, distribution on intervals [0,1], [1,7] and [7,20] with probabilities 0.8, 0.15 and 0.05, respectively; and for the other datasets distribution on intervals [0,1], [1,10] and [10,35] with probabilities 0.9, 0.09 and 0.01, respectively. In all our examples, the coefficients of interference, between adjacent marker segments were simulated as independent random values with uniform distribution on [0,1] or [1,20] intervals, with probabilities 0.85 and 0.15, respectively.

These complications can generate violations of monotony conditions [12]. For a correctly ordered individual map, one would expect that the genetic distance (or recombination rate) from this marker to its adjacent neighbor, and to the next neighbor, etc. will grow monotonically. Deviation from monotony is an indicator of the presence of problematic markers [12, 22]. For building an individual genetic map, we should remove these markers from the skeletal map. However, when the individual maps are constructed as the first phase of consensus mapping analysis of several data sets, such problematic markers of individual genetic map(s) cannot be automatically rejected because a marker may be problematic in some of the maps but not in others. That is why for consensus mapping we use the whole matrix of marker distances for each mapping population participating in the consensus analysis [16] instead of using the accumulated distance between the markers along the map that is used in the approach (i).

## Results

### Testing the algorithm on synthetic genotyping data

The goal was to compare the effectiveness of our three mutation procedures (SCMP, RMP and RCMP) and their combinations. Tables 1 and 2 show the results obtained on datasets Group 1 and Group 2, respectively.

**Table 1:** Testing the proposed algorithm on datasets of Group 1.

Name of dataset	NS	FRS	LCM, cM	*K*_r_	CPU, sec	Number of errors
1	2	3	4	5	6	7
Ex1-1	40.72	161.78	41.30	0.98	4	1
Ex2-1	39.74	157.35	40.20	0.97	4	2
Ex3-1	40.51	160.58	41.10	0.98	4	1
Ex4-1	40.19	161.67	40.64	0.98	8	1
Ex5-1	41.16	162.25	41.85	0.98	5	1
Ex6-1	39.97	159.26	40.58	1.00	3	0
Ex7-1	41.34	157.43	41.78	0.97	3	2
Ex8-1	39.60	154.08	40.13	1.00	2	0
Ex9-1	42.30	160.10	42.86	0.97	3	2
Ex10-1	39.67	158.27	40.28	1.00	4	0

Each dataset of Group 1 contains five subsets of shared markers scored without errors,with different distribution of recombination rates and interference values along the chromosome.

In the table, NS is the sum of lengths of the non-synchronized maps, FRS is the sum of lengths of the initial (random) consensus solution, LCM is the sum of lengths of the optimal consensus maps, and *K*_*r*_ is the coefficient of recovery of the simulated marker order.

**Table 2:** Testing the algorithm on datasets of Group 2.

Name of dataset	NS	FRS	LCM, cM	*K*_r_	CPU, sec	Number of errors
1	2	3	4	5	6	7
Ex1-2	32.5	118.6	32.5	0.79	6	13
Ex2-2	31.7	116.9	31.8	0.87	7	7
Ex3-2	32.9	117.4	32.9	0.84	18	10
Ex4-2	32.7	118.9	32.8	0.87	7	7
Ex5-2	33.2	120.7	33.3	0.86	50	8
Ex6-2	32.3	118.7	32.4	0.90	5	6
Ex7-2	33.2	116.4	33.3	0.86	3	8
Ex8-2	31.8	114.8	31.9	0.87	19	7
Ex9-2	34.5	118.5	34.7	0.84	7	10
Ex10-2	31.4	118.0	31.4	0.84	5	10

Each dataset of Group 2 contains markers of five mapping population, with a total of 100 shared markers scored with errors and missing data point, with different distribution of recombination rates and interference values along the chromosome.

In the table, NS is the sum of lengths of the non-synchronized maps, FRS is the sum of lengths of the first random consensus solution, LCM is the sum lengths of optimal consensus maps, and *K*_*r*_ is the coefficient of recovery of the simulated marker order.

Map quality was evaluated by the coefficient of recovering the true (simulated) marker order *K*_*r*_ (column 5 in Tables 1 and 2) and by the number of errors in marker orders (column 7 in Tables 1 and 2):

$$K_{r}=\left(n_{shared}-1\right)/\sum \left|\left(g_{i}-g_{i}{}_{-1}\right)\right|,$$ 3

where *n*_*shared*_ is the number of shared markers in the dataset; *i* = 2,…, *n*_*shared*_; *g*_*i*_ is number of the marker in position *i* of the solution vector *g*. Computation time for these simulation was 3–8 sec in Group 1 and 3–50 sec in Group 2. The usual type of marker ordering error was one-two inversions of adjacent markers. The effectiveness of the three mutation procedures and their combinations on the simulated problems are presented in S1 Table. The SCMP proved to be the most effective among the three procedures, while their combinations allowed further improvement. In particular, combinations (SCMP+RMP+RCMP) and (SCMP+RMP) demonstrated the highest quality solutions on the tested examples with the same average *K*_*r*_ = 0.955, but the (SCMP+RMP+RCMP) combination was faster (average computation time 8.5 sec against 9.4 sec). The results for the larger size problems (5 sets with 200 and 500 markers) are presented in Table 3.

**Table 3:** The results of consensus mapping on the simulatedproblems of five sets by 200 and 500 shared markers.

Name of dataset	Number of markers	Simulated map length	Global optimization with RMP + SCMP + RCMP	
			LCM	*K*_r_
1	2	3	4	5
Ex3	200	62.9	63.4	0.84
Ex4	500	153.5	154.0	0.89

Datasets Ex3 and Ex4 contain markers scored with errors and missing data, with different distributions of recombination rates and interference values along the chromosome.

In the table, LCM is the sum of lengths of the optimal consensus maps, *K*_*r*_ is the coefficient of recovery of the simulated marker order.

Despite the reading error complications, the optimization algorithm provided high-quality solutions in reasonable computation time. We also tested the effect of using the initial step, i.e., employing the initial solution based on single-set analysis. The efficiency of the initial solutions and the local search procedures on the simulated examples is shown in Table 4. It appeared that performance of the algorithm with the initial solutions was, on average, two-fold higher on the 100-marker problems, and 4- and 12-fold higher on 200- and 500-marker problems, respectively. In addition, we also tested the effect of the “curing” step of each new current best solution (step 7 of the ES algorithm). As one can see from Table 4 (column 5), the utilization of the initial solution step and the local search can considerably reduce the computation time on large-scale problems. It is noteworthy that in our scheme the local search “curing” step is applied only to the new current best solution along the optimization trajectory.

**Table 4:** Comparative effectiveness of the initial solutions and thelocal search procedures on the simulated problems.

Name of dataset	Size of the datasets	CPU time (sec.) to reach the best solution			
		3M^1^ only		Int^2^ + 3M	Int + 3M + LS^3^
1	2	3		4	5
Ex1-1	5×100	6.00		2.30	0.39
Ex2-1	5×100	7.00		0.11	0.40
Ex3-1	5×100	18.00		1.12	0.37
Ex4-1	5×100	7.00		0.55	0.53
Ex5-1	5×100	50.00		12.04	0.42
Ex6-1	5×100	5.00		1.59	0.77
Ex7-1	5×100	3.00		5.11	0.44
Ex8-1	5×100	19.00		13.87	0.39
Ex9-1	5×100	7.00		4.95	0.43
Ex10-1	5×100	5.00		12.08	0.43
Ex1-2	5×100	4.00		1.26	0.42
Ex2-2	5×100	4.00		0.68	0.46
Ex3-2	5×100	4.00		1.31	0.40
Ex4-2	5×100	8.00		0.62	0.44
Ex5-2	5×100	5.00		1,58	0.48
Ex6-2	5×100	3.00		1.93	0.42
Ex7-2	5×100	3.00		2.35	0.40
Ex8-2	5×100	2.00		1.64	0.45
Ex9-2	5×100	3.00		1.80	0.51
Ex10-2	5×100	4.00		1.38	0.44
Average	-	8.35	3.51		0.42
Ex3	5×200	151.00	28.00		1.98
Ex4	5×500	8080.00	680.00		11.70

The utilization of the initial solution step (column 4) and the local search (column 5) considerably reduces the computation time on the test problem.

^1^ The three mutation procedures are working.

^2^ The Initial solution used.

^3^ The local search used.

In the examples provided in S2 and S3 Tables we illustrate how consensus analysis can correct ordering errors in the maps caused by different complications in the data during separate analysis of each population (we denoted such maps as “single-maps”). As one can see from S2 Table, five single-maps of example 6–1 (also present in Table 1) included nine two-marker errors, one three-marker error, one four-marker error, and one five-marker error. The consensus map was error-free with *K*_*r*_ = 1.0. The example 6–2 (presented in Table 2) is more challenging due to genotyping errors. Correspondingly, the obtained consensus map included five two-marker errors (leading to *K*_*r*_ = 0.9 instead of ideal *K*_*r*_ = 1.0); still, this result is a considerable improvement compared to the single-maps that contained 17 two-marker errors, five three-marker errors, three four-marker errors and one five-marker errors (see S3 Table).

### Testing the algorithm on real data

The real data included 17 F_2_ populations of *Arabidopsis thaliana* [21] with all shared SNP markers and known genetic maps. In this data, markers are named according to their position in the chromosomal DNA sequences. We selected chromosome 4 for the test based on separate prior analysis of the 17 datasets for each of the five chromosomes. The number of markers for chromosome 4 varied among populations from 27 to 39 while the total set of shared markers (that appear at least in two populations) was 52. For this chromosome, in 9 out of 17 F_2_ populations the marker order obtained in single-maps differed from the “expected” order (i.e., based on marker position according genome sequence); these local order disturbances included 2–5 markers. Our algorithm resulted in correct marker order for the consensus map (S3 Table).

### Comparing the proposed algorithm with two state-of-the-art algorithms

We compared our algorithm with two consensus mapping solvers, MergeMap [11, 14] and ILPMap [13] which outperform some other widely used software packages (e.g., JoinMap). In order to use the solvers, a pre-compilation step of the original sources was needed. Our input datasets were converted to input format of MergeMap and ILPMap, which uses accumulated marker distances across the maps instead of matrix distance. Note that this data format not enable taking into the information about the violation of monotonic change of recombination rates between markers along the trial map orders (e.g., in orders obtained for individual mapping sets). Quality of the compared algorithms was estimated according to three criteria: number of order errors in the maps, number of markers in the error zone, and required CPU. On the 22 tested problems, our algorithm outperformed both MergeMap and ILPMap in 11 problems and in 7 problems it returned the same results as the best of the two competitors (Table 5). Only for two problems out of the 22 (EX2-1 and EX7-1) ILPMap produced slightly better results than those obtained by our and MergeMap algorithms. On the tested problems, ILPMap outperforms MergeMap in 13 problems, but it did not solve the problems EX10-1 and EX7-2 at all. MergeMap was better than ILPMap for 5 out of the 22 test problems. It is noteworthy that the solutions by our algorithm included errors of only adjacent markers, while errors in MergeMap and ILPMap solutions included also error zones of 3–6 markers. The most difficult for ILPMap was the EX3 problem in which only 60 markers from 200 appeared in unambiguously correct order. The remaining 140 markers were present as bins with 2–10 not mutually ordered markers.

For an additional comparison, we employed real data on *Arabidopsis* [21]. Consensus mapping for this data was performed using three algorithms (our, MergeMap and IPLMap). Our algorithm and MergeMap resulted in true marker order for the consensus map, but CPU of MergeMap was 200 seconds vs one second of our algorithm. ILPMap produced the consensus map with only one error marker order (reverse order of markers m17 and m18).

**Table 5:** Comparing the efficiency of three consensus mapping algorithms.

Number of Problem	Synchronized-TSP			ILPMap			MergeMap		
	Errors	*K*_*r*_	CPU, sec	Errors	*K*_*r*_	CPU, sec	Errors	*K*_*r*_	CPU, sec
1	2	3	4	5	6	7	8	9	10
Ex1-1	1	0.98	0.39	1	0.98	1.0	4	0.92	357
Ex2-1	2	0.97	0.40	**1**	**0.98**	1.0	7	0.87	38
Ex3-1	1	0.98	0.37	1	0.98	1.0	6	0.88	102
Ex4-1	1	0.98	0.53	1	0.98	1.0	6	0.88	132
Ex5-1	**1**	**0.98**	0.42	7	0.87	1.0	12	0.80	240
Ex6-1	**0**	**1.00**	0.77	4	0.92	1.0	5	0.90	90
Ex7-1	2	0.97	0.44	**0**	**1.00**	1.0	2	0.96	87
Ex8-1	0	1.00	0.39	0	1.00	1.0	5	0.90	94
Ex9-1	**2**	**0.97**	0.43	4	0.92	1.0	9	0.85	70
Ex10-1	**0**	**1.00**	0.43	na^3^	-	-	9	0.85	60
Ex1-2	**13**	**0.79**	0.42	15	0.70	1.0	14	0.73	6
Ex2-2	7	0.87	0.46	7	0.87	1.0	7	0.87	280
Ex3-2	10	0.84	0.40	10	0.84	1.0	14	0.73	2
Ex4-2	7	0.87	0.44	7	0.87	1.0	8	0.86	7
Ex5-2	8	0.86	0.48	8	0.86	1.0	14	0.73	29
Ex6-2	6	**0.90**	0.42	6	0.88^2^	1.0	8	0.86	21
Ex7-2	**8**	**0.86**	0.40	na^3^	-	-	11	0.82	7
Ex8-2	**7**	**0.87**	0.45	10	0.84	1.0	9	0.85	3
Ex9-2	10	0.84	0.51	10	0.84	1.0	10	0.84	1
Ex10-2	10	0.84	0.44	11	0.82	1.0	10	0.84	5
Ex3	**15** ^1^	**0.84**	1.98	55^2^	0.51	3.0	20^2^	0.78	40
Ex4	**23** ^1^	**0.89**	11.70	48^2^	0.75	10.0	38	0.80	270
Real data	**0**	**1.00**	**1.0**	1	0.96	5.0	**0**	**1.0**	200
**Average**	**5.8**	**0.92**	**1.03**	9.8	0.873	1.65	9.9	0.85	93.1
***p*-value** ^**4**^	**-**	**-**	**-**	0.008	0.015	0.0005	0.0002	0.0002	0.00003

^1^ Two adjacent markers in the erroneous order.

^2^ Sequence of 3–6 markers in the erroneous order.

^3^ Not available: no solution was returned by ILPMap.

^4^ By comparing to Synchronized-TSP using Wilcoxon [26] matched pairs test.

## Conclusions

New chip-based platforms for scoring SNP markers bring several positive aspects in consensus mapping. Firstly, chip analysis allows producing high-density genetic maps with a relatively standardized set of marker loci for different mapping populations. The availability of common standard mapping platform attenuates the need in dealing with a considerable proportion of unique (population specific) markers as a part of the optimization problem in cases of insufficient density of shared markers. The ever-increasing number of markers available by chip technology enables ignoring unique markers at the stage of consensus mapping, thereby reducing mathematical complicity of consensus analysis and solving bigger size problems using global optimization criteria instead of the local ones. For mapping problems with thousands markers per chromosome, our three-phase analytical scheme includes: automatic selection of ~100–300 of the most informative resolvable by recombination markers per linkage group [19, 20]; building a stable skeletal marker order for each data set [22, 23]; and conducting consensus mapping by the algorithm proposed here with the global optimization criterion.

During the optimization, the ES algorithm generates possible consensus marker orders via three types of parallel mutation procedures (RMP, SCMP and RCMP) and then tries them by the criterion (1). SCMP and RCMP generate marker orders as random voting on a restricted area of possible marker orders presented in the list of marker neighbors *L*_*ij*_. These two new procedures (SCMP and RCMP) significantly accselerate the optimization process while the local search with a small neighborhood is able to cure the generated maps along conflicted and non-conflicted regions. RMP generates random combinations of markers on the full set of possible marker orders *G*. This allows trying marker orders that are not present in the the single-population maps.

Our algorithm based on ES optimization was tested on different large-scale consensus genetic mapping problems. The best results were achieved by cooperative working of the three mutation procedures described in this paper and employing the initial solution step. A (1/*λ*)-selection strategy instead of the recently applied (1+1)-strategy [16] increases the diversity of solutions generated by the mutation procedures. These new possibilities, applied to problems where all markers are shared at least by two mapping populations, significantly accelerate the Evolution Strategy algorithm [9, 16], and allow solving consensus genetic mapping problems by the global optimization criteria and/or of considerably higher dimensionality. In general, according to the tests performed on the 23 problems, we can conclude that our algorithm, which is especially suitable for SNP datasets with a low proportion of unique markers, outperforms both MergeMap and ILPMap by accuracy and computer time time (by using Wilcoxon [26] matched pairs test). The proposed analytical scheme is able to generate high-quality, ultra-dense consensus maps, with thousands of markers per genome. Based on our tests, an empirical estimate of complexity of the new ES algorithm is well approximated by ~*O*(*n* ^2^). The program implementing the new consensus mapping algorithm and the data for the employed 23 tests can be downloaded using the link http://evolution.haifa.ac.il/images/stories/Software/MultiPointConsensus_DemoG.rar. Short manual to use the ultra-dense consensus mapping program for analyzing the 23 tests is represented in file S2 Text.

New genotyping-by-sequencing (GBS) technologies ([27, 28]) based on massive parallel sequencing yield genotyping data from up to a million sites within a mapping population, but at the cost of high rates of missing data per individual and high proportion of markers with heavily disturbed segregations. A further complication is that the percentage of markers shared across mapping populations will vary depending on the specific characteristics of the selected GBS technology and the genetic relatedness of the populations. Our new algorithm for ultra-dense mapping implemented in MultiPointULD software proved efficient in analysis of individual populations genotyped using GBS or chip-based technologies [19, 20]. However, despite this solution for ultra-dense mapping in single-population situations, the noted complications in GBS data imply a low proportion of high-quality shared markers, calling for an extension of the consensus mapping approach described here to enable an efficient analysis of such data. One of the perspectives would be an additional step that will allow using the constructed consensus maps for shared markers as anchors for positioning of population-specific (unique) markers.

## Supporting Information

S1 Table Comparative effectiveness of combination of the three mutation procedures (SCMP, RMP and RCMP) on the datasets of Group 1, 2. The SCMP proved to be the most effective among the three procedures (*K*_*r*_ = 0.950); combinations (SCMP+RMP+RCMP) and (SCMP+RMP) demonstrate highest quality solutions, with the same average *K*_*r*_ = 0.955 on the tested examples.
(DOCX)

S2 Table Comparing marker order of the original and consensus maps (Example 6–1). ^1^Wrong local marker orders are marked in black.
(DOCX)

S3 Table Comparing marker order of the original and consensus maps (Example 6–2). ^1^ Wrong local marker orders are marked in black.
(DOCX)

S4 Table Comparing marker order of the original and consensus maps (real data). ^1^ Wrong local marker orders are marked in black.
(DOCX)

S1 Text Evolution Strategies for solving combinatorial optimization problems. (DOC)

S2 Text Short manual to use the ultra-dense consensus mapping program for analyzing the 23 tests. (PDF)
