# Supplementary material for: Fast and Accurate Construction of Ultra-Dense Consensus Genetic Maps Using Evolution Strategy Optimization
Source: PLoS One. 2015 Apr 13;10(4):e0122485. doi: 10.1371/journal.pone.0122485 (PMC4395089; doi:10.1371/journal.pone.0122485)
Supplement: S1 Table — The SCMP proved to be the most effective among the three procedures (K r = 0.950); combinations (SCMP+RMP+RCMP) and (SCMP+RMP) demonstrate highest quality solutions, with the same average K r = 0.955 on the tested examples. (DOCX) [file pone.0122485.s001.docx]

**S1 Table. Comparative effectiveness of combination of three mutation procedures**

**(SCMP, RMP and RCMP) on 20 tests of Groups 1 and 2.**

| Type of mutation  procedures | RMP | SCMP | RCMP | RMP +  SCMP | SCMP +  RCMP | RMP +  RCMP | RMP +  SCMP +  RCMP |
| --- | --- | --- | --- | --- | --- | --- | --- |
| Average coefficient of  of recovering, *K_r_* | 0.746 | 0.950 | 0.504 | **0.955** | 0.952 | 0.921 | **0.955** |

The SCMP proved to be the most effective among the three procedures (*K_r_*=0.950); combinations (SCMP+RMP+RCMP) and (SCMP+RMP) demonstrate highest quality solutions, with the same average *K_r_*=0.955 on the tested examples.
