# Supplementary material for: Fast and Accurate Construction of Ultra-Dense Consensus Genetic Maps Using Evolution Strategy Optimization
Source: PLoS One. 2015 Apr 13;10(4):e0122485. doi: 10.1371/journal.pone.0122485 (PMC4395089; doi:10.1371/journal.pone.0122485)
Supplement: S2 Table — 1Wrong local marker orders are marked in black. (DOCX) [file pone.0122485.s002.docx]

**S2 Table.** **Comparing marker order of the original and consensus maps**

**(Example 6-1).**

| Set number | Marker order in the original maps^1^ |
| --- | --- |
| 1 | 2 4 6 8 10 12 14 16 18 20 22 24 26 28 30 32 34 36 38 40 42 44 46 48 50 52 54 56 58 60 62 64 66 68 70 72 74 76 78 80 82 84 86 88 90 92 94 96 98 100 |
| 2 | 1 3 5 7 9 11 13 15 17 19 21 23 25 27 29 31 33 35 37 39 41 43 45 47 49 51 53 55 57 59 61 63 65 67 69 71 75 73 77 79 81 83 85 87 89 91 93 95 97 99 |
| 3 | 2 4 6 8 14 16 12 10 18 20 22 24 26 28 30 32 34 36 42 38 40 44 46 48 50 52 54 56 58 60 62 64 66 70 68 72 74 76 78 80 82 84 86 88 90 92 94 96 98 100 |
| 4 | 1 3 5 7 9 11 13 15 17 19 23 21 25 29 27 31 33 35 37 39 41 43 45 47 49 51 53 55 57 59 61 63 65 67 69 71 73 75 77 79 81 83 85 87 89 91 93 95 97 99 |
| 5 | 1 3 2 4 6 5 7 8 9 10 11 12 13 14 15 16 17 18 19 21 20 22 23 24 25 30 29 28 27 26 31 32 33 34 35 36 37 38 39 40 41 42 43 44 45 46 47 48 49 50 51 52 53 54 55 56 57 58 59 60 61 62 63 64 65 66 67 68 69 70 71 72 73 74 75 76 77 79 78 80 82 81 83 84 85 86 87 88 89 90 91 92 93 94 95 96 97 98 99 100 |
| Consensus | 1 2 …100 (no errors in ordering, *k_r_*=1.0) |

^1^Wrong local marker orders are marked in black.
