# Supplementary material for: Fast and Accurate Construction of Ultra-Dense Consensus Genetic Maps Using Evolution Strategy Optimization
Source: PLoS One. 2015 Apr 13;10(4):e0122485. doi: 10.1371/journal.pone.0122485 (PMC4395089; doi:10.1371/journal.pone.0122485)
Supplement: S3 Table — 1 Wrong local marker orders are marked in black. (DOCX) [file pone.0122485.s003.docx]

**S3 Table. Comparing marker order of the original and consensus maps**

**(Example 6-2).**

| Set number | Marker order in the original maps^1^ |
| --- | --- |
| 1 | 2 4 6 8 10 12 14 16 18 20 22 24 26 28 30 32 34 36 38 40 42 44 46 48 50 52 54 56 58 60 62 64 66 68 70 72 74 76 78 80 82 84 86 88 90 92 94 96 98 100 |
| 2 | 1 3 5 7 9 11 13 15 17 19 21 23 25 27 29 31 33 35 37 39 41 43 45 47 49 51 53 55 57 59 61 63 65 67 69 71 75 73 77 79 81 83 85 87 89 91 93 95 97 99 |
| 3 | 1 2 3 5 4 6 7 8 9 11 10 12 13 14 16 15 17 18 19 20 21 22 23 25 24 26 27 28 29 31 32 33 34 35 36 37 38 40 39 42 41 43 44 45 46 47 48 49 50 51 52 54 55 56 53 57 58 59 60 61 62 6364 65 66 67 68 69 70 71 72 73 74 75 76 77 78 79 80 81 82 83 84 85 86 87 88 90 89 91 92 93 94 95 96 97 98 99 100 |
| 4 | 1 2 4 5 3 6 7 8 9 11 10 12 13 14 17 15 16 18 19 20 22 21 23 24 25 26 29 27 28 30 31 32 35 34 33 38 36 37 39 40 42 43 41 44 45 46 47 48 49 50 52 51 53 54 55 56 57 59 58 60 61 62 63 64 65 68 67 66 69 70 71 72 73 75 74 76 77 78 79 80 82 83 84 81 85 86 87 88 90 89 91 92 93 94 95 96 97 98 99 100 |
| 5 | 1 3 2 4 6 5 7 8 9 10 11 12 13 14 15 16 17 18 19 21 20 22 23 24 25 30 29 28 27 26 31 32 33 34 35 36 37 38 39 40 41 42 43 44 45 46 47 48 49 50 51 52 53 54 55 56 57 58 59 60 61 62 63 64 65 66 67 68 69 70 71 72 73 74 75 76 77 79 78 80 82 81 83 84 85 86 87 88 89 90 91 92 93 94 95 96 97 98 99 100 |
| Consensus | 1 3 2 4 5 6 7 8 9 10 11 12 13 14 15 16 17 18 19 21 20 22 23 24 25 27 26 28 29 30 31 32 33 34 35 36 37 38 39 40 41 42 43 44 45 46 47 48 49 50 51 52 53 54 55 56 57 58 59 60 61 62 63 64 65 66 67 68 69 70 71 72 73 74 75 76 77 79 78 80 82 81 83 84 85 86 87 88 89 90 91 92 93 94 95 96 97 98 99 100 |

^1^ Wrong local marker orders are marked in black.
