# Supplementary material for: Fast and Accurate Construction of Ultra-Dense Consensus Genetic Maps Using Evolution Strategy Optimization
Source: PLoS One. 2015 Apr 13;10(4):e0122485. doi: 10.1371/journal.pone.0122485 (PMC4395089; doi:10.1371/journal.pone.0122485)
Supplement: S4 Table — 1 Wrong local marker orders are marked in black. (DOCX) [file pone.0122485.s004.docx]

**S4 Table. Comparing marker order of the original and consensus maps (real data).**

| Set  number | Number of markers | Order of the markers^1^ |
| --- | --- | --- |
| 1 | 39 | m1 m2 m3 m4 m8 m6 m5 m10 m9 m11 m13 m15 m16 m17 m19 m20 m21 m22 m24 m25 m26 m27 m29 m32 m33 m34 m35 m36 m40 m41 m42 m43 m44 m45 m46 m47 m48 m51 m52 |
| 2 | 39 | m2 m3 m4 m8 m7 m5 m6 m9 m10 m11 m13 m15 m16 m19 m21 m22 m24 m25 m27 m28 m30 m31 m32 m33 m34 m35 m36 m37 m40 m41 m42 m43 m44 m46 m47 m48 m49 m50 m51 |
| 3 | 32 | m1 m2 m3 m4 m5 m8 m9 m16 m17 m19 m20 m22 m24 m25 m26 m28 m30 m31 m33 m34 m35 m37 m38 m39 m40 m42 m43 m45 m46 m47 m49 m50 |
| 4 | 31 | m2 m3 m8 m7 m10 m5 m6 m11 m13 m20 m21 m22 m24 m26 m27 m29 m31 m32 m34 m36 m37 m38 m41 m44 m45 m46 m48 m49 m50 m51 m52 |
| 5 | 35 | m1 m2 m3 m4 m5 m6 m7 m11 m13 m15 m16 m17 m19 m20 m23 m24 m27 m28 m30 m32 m33 m36 m37 m38 m39 m40 m41 m42 m43 m44 m46 m47 m50 m49 m52 |
| 6 | 34 | m1 m2 m4 m6 m5 m7 m12 m15 m17 m18 m20 m21 m23 m25 m26 m27 m28 m29 m31 m33 m34 m36 m37 m38 m39 m40 m41 m43 m45 m47 m49 m50 m51 m52 |
| 7 | 33 | m1 m3 m4 m5 m6 m8 m9 m10 m11 m13 m19 m20 m21 m22 m23 m24 m25 m26 m28 m30 m31 m32 m33 m36 m37 m38 m42 m44 m45 m47 m48 m50 m51 |
| 8 | 36 | m2 m3 m4 m7 m8 m9 m10 m11 m14 m15 m16 m17 m19 m21 m22 m24 m27 m28 m29 m30 m31 m33 m34 m35 m37 m39 m41 m42 m44 m45 m46 m48 m49 m50 m51 m52 |
| 9 | 37 | m1 m2 m3 m4 m6 m7 m9 m10 m11 m12 m13 m16 m17 m21 m23 m24 m26 m28 m29 m30 m31 m33 m34 m35 m36 m37 m38 m40 m41 m43 m44 m45 m47 m49 m50 m51 m52 |
| 10 | 32 | m1 m2 m5 m7 m8 m9 m10 m13 m15 m16 m17 m20 m21 m23 m25 m27 m28 m29 m30 m32 m34 m37 m38 m39 m42 m43 m44 m45 m46 m48 m49 m51 |
| 11 | 30 | m2 m3 m4 m13 m16 m17 m19 m20 m21 m22 m23 m25 m29 m30 m31 m33 m34 m35 m36 m38 m39 m42 m43 m44 m47 m48 m49 m50 m51 m52 |
| 12 | 31 | m1 m4 m6 m7 m10 m9 m11 m13 m16 m17 m19 m21 m25 m27 m28 m29 m30 m32 m33 m35 m36 m39 m40 m41 m42 m43 m44 m47 m48 m51 m52 |
| 13 | 31 | m2 m3 m5 m7 m8 m12 m9 m15 m16 m17 m19 m20 m23 m25 m27 m29 m31 m32 m34 m35 m36 m37 m38 m39 m40 m42 m43 m46 m50 m51 m52 |
| 14 | 27 | m1 m2 m6 m9 m12 m15 m17 m19 m20 m22 m23 m27 m29 m31 m33 m35 m36 m37 m39 m41 m43 m44 m45 m46 m48 m49 m51 |
| 15 | 32 | m1 m3 m7 m6 m9 m13 m15 m17 m18 m20 m21 m23 m24 m27 m28 m29 m33 m34 m35 m36 m37 m38 m39 m40 m41 m43 m44 m46 m47 m48 m49 m52 |
| 16 | 35 | m1 m3 m5 m6 m7 m13 m14 m15 m16 m17 m18 m19 m20 m21 m22 m24 m27 m28 m29 m30 m34 m35 m36 m37 m38 m39 m40 m42 m44 m45 m46 m47 m49 m50 m51 |
| 17 | 35 | m2 m4 m8 m5 m10 m11 m13 m14 m15 m16 m18 m19 m20 m21 m22 m24 m25 m26 m31 m32 m33 m34 m35 m36 m38 m40 m41 m42 m44 m45 m46 m47 m49 m51 m52 |
| Consensus map | 52 | m1 m2…m51 m52 (no errors in ordering, *k_r_*=1.0) |

^1^ Wrong local marker orders are marked in black.
