# Supplementary material for: Fast and Accurate Construction of Ultra-Dense Consensus Genetic Maps Using Evolution Strategy Optimization
Source: PLoS One. 2015 Apr 13;10(4):e0122485. doi: 10.1371/journal.pone.0122485 (PMC4395089; doi:10.1371/journal.pone.0122485)
Supplement: S1 Text — (DOC) [file pone.0122485.s005.doc]

Evolution Strategies for solving combinatorial optimization problems

ES is a heuristic algorithm mimicking natural population processes. The numerical procedures in such optimization are based on simulation of mutation, followed by selection of the fittest “genotypes” founded on obtained values of the optimization criterion. In contrast to GA, ES does not employ recombination; mutation is the only mechanism to change a solution in ES optimization algorithm. Clearly, combinatorial problems cannot be directly represented in terms of ES with real-value formulation. Combinatorial versions of ES differ from the real-value formulation by a specific representation of the solution vector *x* and the mutation mechanisms. In a combinatorial formulation, a solution to a discrete optimization problem can be represented as a vector *x* = (*x*_1_, *x*_2_,*...*, *x_n_*) with *n* ranked discrete coordinates or as directed graph *V*(*A, B*) with a set of nodes *A* = {*a*_1_, *a*_2_, *...a_n_*} and a set of arcs *B*=*A*×*A*. The *mutation operator* (referred to hereafter as *mutator*) changes the order of some components of vector *x^k^* thereby producing a new solution vector *x^k^*^+1^. The fitness function assigns to each arc (*a_i_*, *a_j_*) or pair of coordinates (*x_i_*, *x_j_*) of the solution vector *x^k^*^+1^ a non-negative parameter *d_ij_*, the cost of moving from element *i* to element *j*. For combinatorial optimization, one needs to define such an order of the vector coordinates (or nodes) that will provide minimum total cost *f*(*x*). If after the current selection step *f*(*x^k^*^+1^) is better than *f*(*x^k^*), then the optimization process will continue with the new vector *x^k^*^+1^. In contrast to such a process with population size *λ=*1, various approaches were proposed with *λ*>1 allowing to consider selection strategies defining how many *μ*≥1 and which best solutions should be taken for the next generation [1-4].

One of the central questions in ES algorithms concerns the *mutation strategies*. Three components of mutation strategy have to be defined for an ES algorithm. The first component mimics the appearance of mutation and can be referred to as *mutation procedure*. For that, one can use *move-generation* and *solution-generation* procedures [5, 6]. The *move-generation* procedure changes position of some components of solution vector by a *move* procedure, for example: *Exchange* (it exchanges the positions of two random components), 2-*Opt move* (reverses all components between two random components), *Reinsert* (shifts some random components in small neighborhood to another random position in the solution vector; [7]) and 2-Opt procedures [8]. The procedures mimic natural processes where mutation and recombination may depend on environment and organism’s fitness [9, 10]. The second component forms the neighborhood to be targeted by mutation mechanism. Optimal neighborhood size is very important for effective solving large-scale problems. Local search on large neighborhoods increases the executing time; small neighborhoods accelerate the optimization process but cannot perturb remote points on large solution vectors [12]. The *Variable Neighborhood* strategy, proposed by [11, 12], combines ideas of large and small neighborhoods approaches in such a way that smaller variable neighborhoods are generated more often than large ones. Some versions of variable neighborhood, namely *Adaptive Variable Neighborhood* and *Penalty Variable Neighborhood*, were developed for different vehicle routing problems [13-15] and for genome mapping problems [12, 16]. The third component defines the mutation size on the selected neighborhood, i.e., how many components in the solution vector will change their positions. This is the *remove* step in the “remove-insert” mutation mechanism. In ES algorithms, usually small mutation disturbances to the solution vector are desirable. We found no clear formulation of the notion *small mutation* for the discrete optimization problem in the earlier literature. Consequently, we attempted to provide such a formulation together with the notion of *Variable Mutation Size* that is defined as the number of removed points Ω (in our algorithm Ω can be up to 70% of the solution vector) [11]

Ω = (0*.*2 + 0*.*5*f* ^2^) *n,* (1)

where *n* is the number of vector components in the variable neighborhoods and *f* is random value uniformly distributed between 0 and 1. In addition, with a small probability (e.g., 0.01), we set *Ω* = *n*. Such relatively high mutation rate compared to the usually employed 5-10% [1, 2] is useful in the subsequent ‘*curing*’ of the variable neighborhoods via a simple local search. *Curing* is a stage of evolution strategy analogous to some natural processes, but it has not been considered thus far in the discrete optimization literature as an essential element of ES. The meaning of the second and third mutation components is well coordinated with the known H.-P. Schwefel‘s opinion: "Smaller mutations must occur more often than larger ones" (cited by [17]). In general, multi-parametric mutation mechanism for ES discrete optimization algorithms can be defined as an operator which transforms *x^k^* into *x^k^*^+1^:

*x^k^*^+1^ = M{*x^k^*, *α*, *β*, **γ** } (2)

The set of parameters for *α*, *β* and *γ* defines concrete multi-parametric mutation mechanism in ES algorithms for discrete optimization problems. In our recent ES algorithm [18], a random multi-parametric mutation procedure and (1+1)-selection strategy were used. The new ES algorithm for the proposed here hybrid approach is strengthened by new optimization and selection strategies compared to our previous version of the algorithm [16, 18].

**References**

1. Rechenberg I. Evolutionstrategie. Stuttgart: Romman-Holzboog Press;1973.
2. Schwefel H-P. Numeriche optimierung von computer-modellen mittels der evolutions strategie. Basel: Birkauser Press; 1977.
3. Gehring H, Homberger J. A parallel hybrid evolutionary metaheuristic for the vehicle routing problem with time windows. In: Proceedings of EUROGEN99, Jyvaskyla, University of Jyvaskyla; 1999. pp. 57-64.
4. Mester D, Ronin Y, Minkov D, Nevo E, Korol A. Constructing large-scale genetic maps using an evolutionary strategy algorithm. Genetics 2003; 165: 2269-2282.
5. Osman IH. Metasrategy simulated annealing and tabu search algorithm for VRP. Annals of Operation Research 1993; 41: 421-451.
6. Homberger J, Gehring H. Two evolutionary metaheuristics for vehicle routing problem with time windows. INFOR 1999; 37: 297-318.
7. Flood MM. The traveling-salesman problem. Operation Research 195; 4: 61-75.
8. Or I. Traveling salesman-type combinatorial problems and their relations to the logistics of region, blood banking. Ph.D. Thesis, Department of Industrial Engineering and Management Science, North Western University, USA.1976.
9. Korol AB. Recombination. In: Levin SA, editor. Encyclopedia of Biodiversity, 2nd edition, Vol. 6, Waltham: Academic Press; 2013. pp. 353-369.
10. Korol AB, Preygel IA, Preygel SI. Recombination variability and evolution*.* London: Chapman & Hall; 1994.
11. Mester D, Ronin Y, Minkov D, Nevo E, Korol A. Constructing large-scale genetic maps using an evolutionary strategy algorithm. Genetics 2003; 165**:** 2269-2282.
12. Mester D, Ronin Y, Nevo E, Korol A. Fast and high precision algorithms for optimization in large scale genomic problems*.* Comp Biol & Chemistry 2004; 28: 281-290.
13. Mester D, Bräysy O. Active guided evolution strategies for large scale vehicle routing problems with time windows. Comp Oper Res 2005; 32: 1593−1614.
14. Mester D, Bräysy O. Active guided evolution strategies for large scale capacitated vehicle routing problems. Comp Oper Res 2006; 34: 2964-2975.
15. Mester D, Bräysy O, Dulaert W. A multi-parametric evolution strategies algorithm for vehicle routing problems. Expert Systems with Application 2007; 32: 508-717.
16. Mester D, Ronin Y, Korostishevsky M, Frenkel Z, Bräysy O, Dullaert W, et. al. Discrete optimization for some TSP-like genome mapping problems. In: Varela J, Acuna S, editors. Operations Research: Decision Analysis, Optimization, and Applications. New York: Nova Science Publishers; 2010. pp. 1-41.
17. Bäck T. Evolutionary algorithms in theory and practice. New York: Oxford university Press; 1996.
18. Ronin Y, Mester D, Minkov D, Belotserkovski R, Jackson B, Schnable P, et al. Two-phase analysis in consensus genetic mapping. G3 2012; 5: 537-549.
