# Supplementary material for: Fast and Accurate Construction of Ultra-Dense Consensus Genetic Maps Using Evolution Strategy Optimization
Source: PLoS One. 2015 Apr 13;10(4):e0122485. doi: 10.1371/journal.pone.0122485 (PMC4395089; doi:10.1371/journal.pone.0122485)
Supplement: S2 Text — (PDF) [file pone.0122485.s006.pdf]

Short manual

to use the ultra-dense consensus mapping program for analyzing the 23 tests (employed in the manuscript by Mester et al. “Fast and accurate construction of ultra-dense consensus genetic maps using evolution strategy optimization”)

Introduction

First, we would like to note that the program is not a demo of a user-friendly software package; it only demonstrates the ultra-dense consensus mapping algorithm (described in the paper) on a set of 23 examples without providing numerous service functions expected in a software package.

The algorithm was implemented in Visual Basic 6 under Windows 7 and tried on a desktop PC (4-core 2.8GHz Intel Pentium processor and 8GB RAM). Each data file of the tested examples and the program file “LM-ES-3G -Q12-VrLimCompvarA1.exe” are placed in a single sub-folder. For the program, standard names “inputsynchro.txt” and “outputsynchro.txt” for input and output files, respectively, are used for each example. The structure of the files is described below.

System requirements are as follows:
- PC 32 or 64 bit under Windows 7;
- MSWord editor from MSOffice - 2010 (by default the MSWord produces *.docx files); Adobe Reader 11 (Adobe security functions =OFF);
- Standard **Microsoft 7-zip File Manager** (that decompresses *.rar and many others compressed files automatically).The archiver is available in http://www.7-zip.org/download.html ;
- Our default browser is **Google Chrome**.

For using the program and the datasets do the following:

1. **Decompress** folder “MultiPointConsensus_DemoG.rar”

   This folder contains two subfolders:
   - “Examples and program”;
   - “Installation Package”.

1.1. Ctrl-Click the link

http://evolution.haifa.ac.il/images/stories/Software/MultiPointConsensus_DemoG.rar

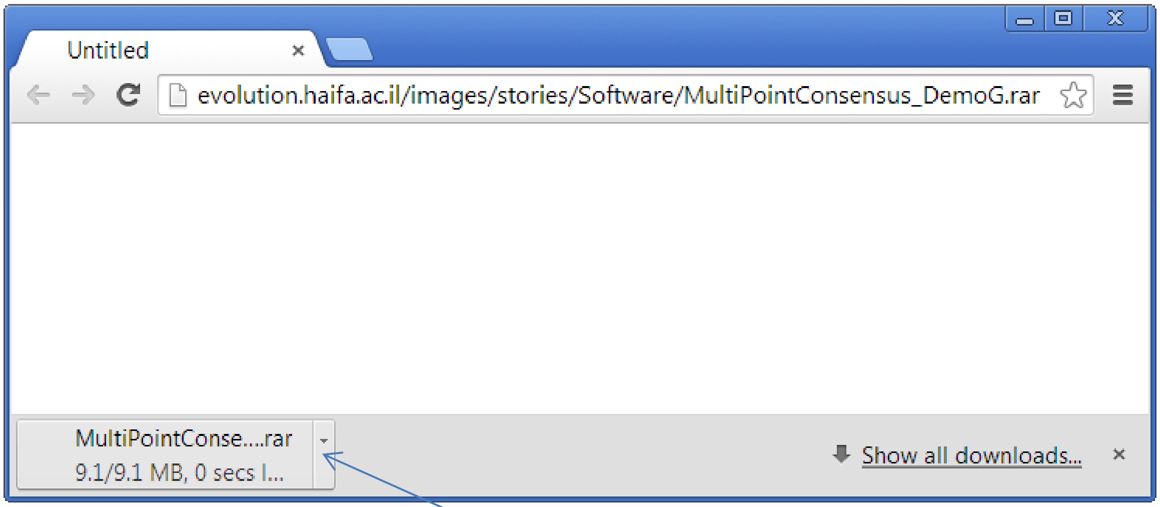

1.2. Google Chrome downloads the "MultiPointConsensus_DemoG.rar" file to the customer computer. To see the downloaded file, click the button and then click "Show in folder" options in the appeared sub-window.

1.3. Drag (by mouse) the "MultiPointConsensus_DemoG.rar" file to the PC Desktop

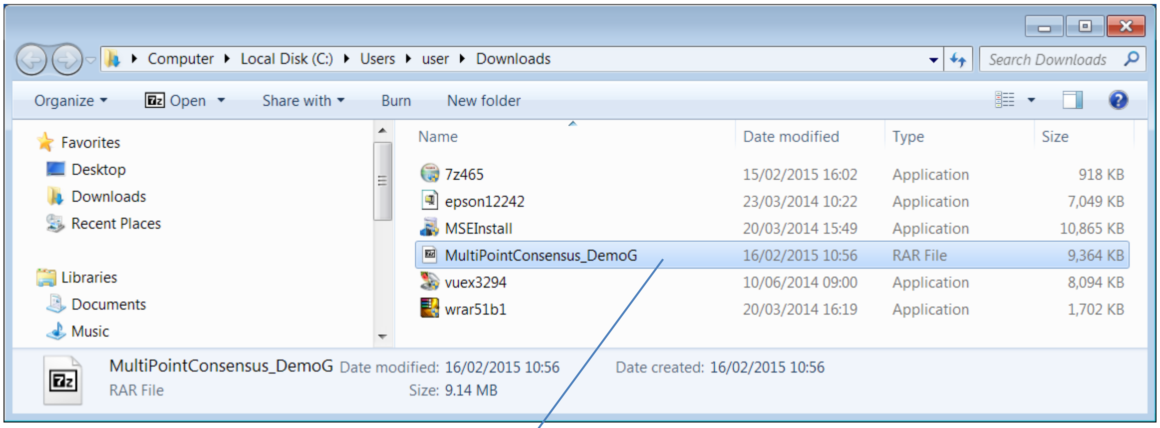

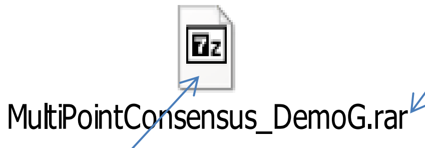

1.4. Click the obtained icon for decompressing of the downloaded *.rar file.

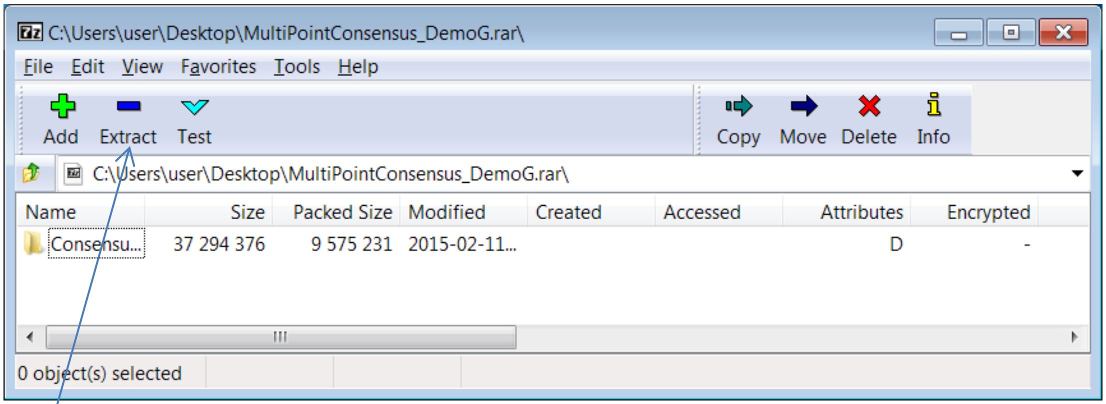

1.5. **Extract** the compressed folder. **MS 7-zip File Manager** will create decompressed folder “Consensus_mapping" on the PC Desktop**.**

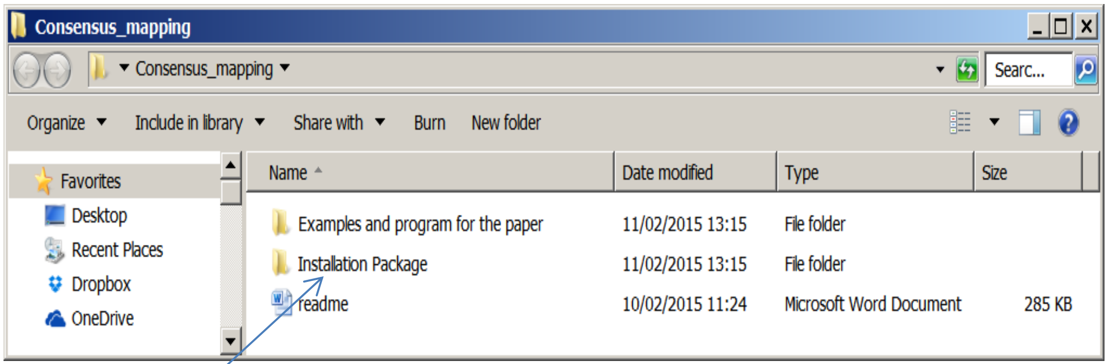

2. Install the program

For using the consensus mapping program, **Run** the Setup.exe file in the **Installation Package** folder.

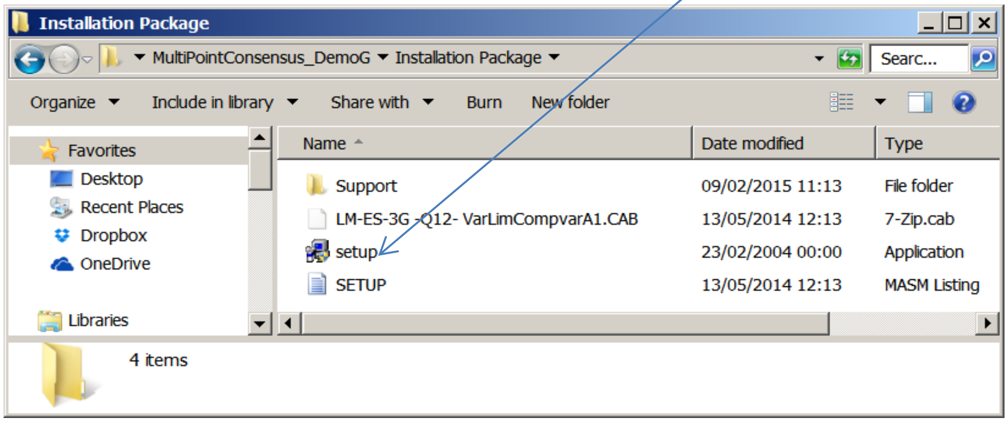

As a result, the **GKsetup** installation program will install the consensus mapping program modules into Windows system. During the installation process, the following screen shots (2.1 – 2.4) will appear:

2.1.

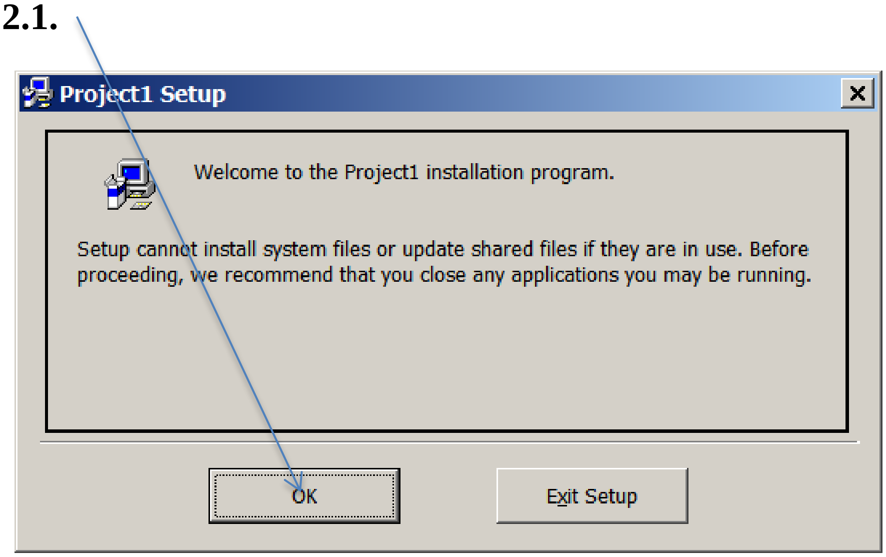

2.2.

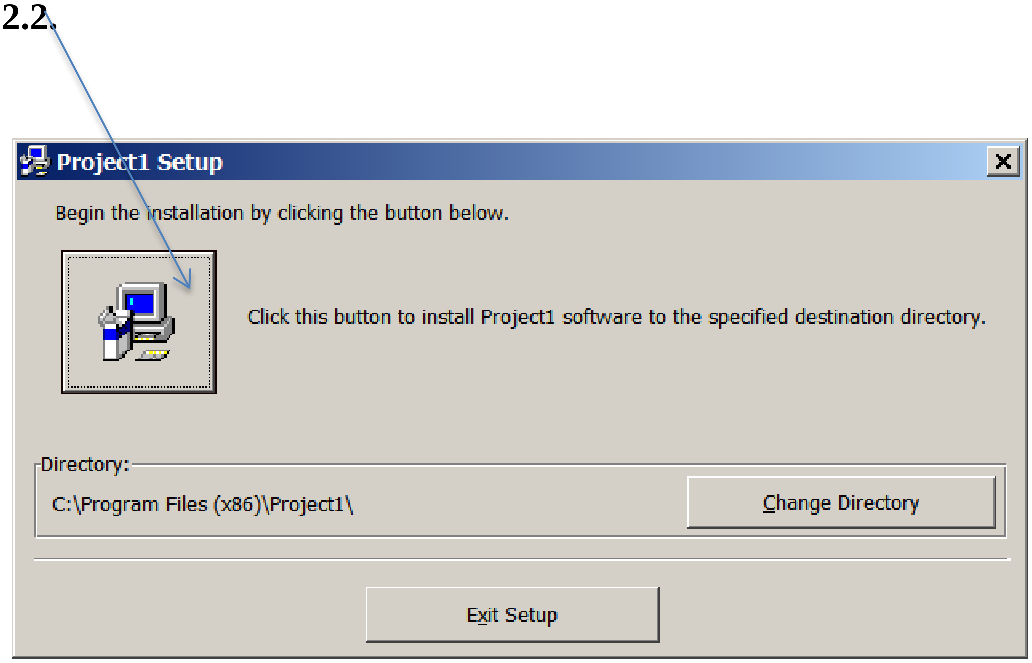

2.3.

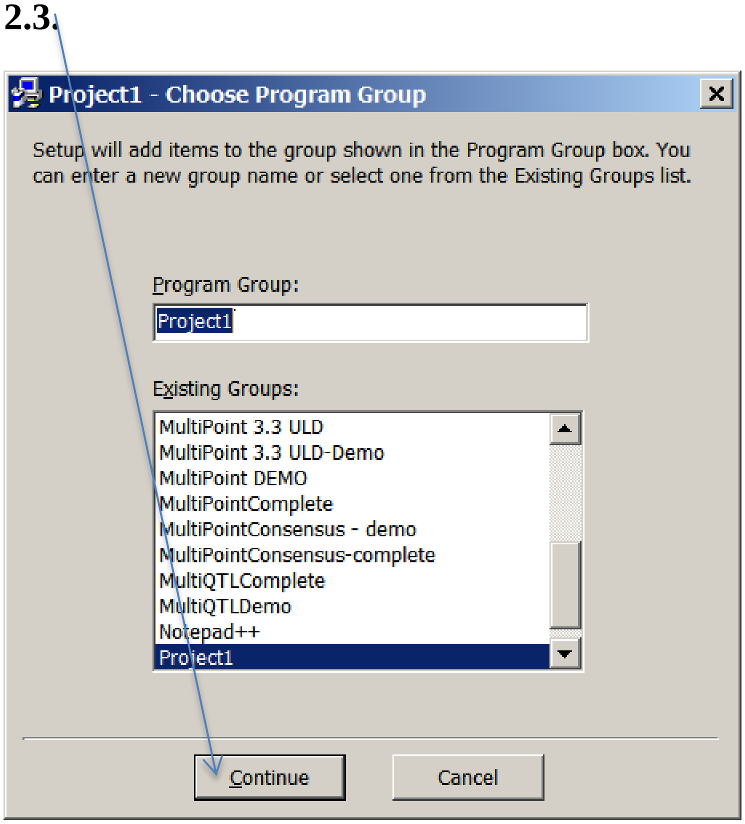
Project1 - Choose Program Group
Setup will add items to the group shown in the Program Group box. You can enter a new group name or select one from the Existing Groups list.
Program Group:
Project1
Existing Groups:
MultiPoint 3.3 ULD
MultiPoint 3.3 ULD-Demo
MultiPoint DEMO
MultiPointComplete
MultiPointConsensus - demo
MultiPointConsensus-complete
MultiQTLComplete
MultiQTLDemo
Notepad++
Project1
Continue
Cancel

2.4.

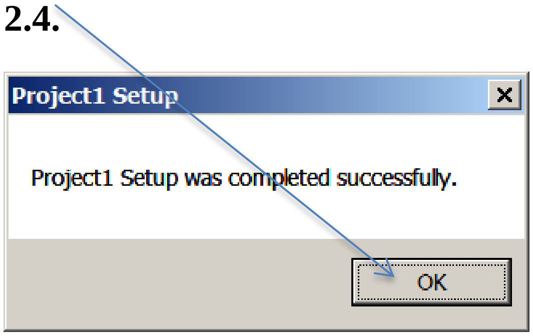
Project1 Setup
Project1 Setup was completed successfully.
OK

3. **Start** the program on the example datasets.

Each example runs in its own subfolder of “Examples and program for the paper” folder.

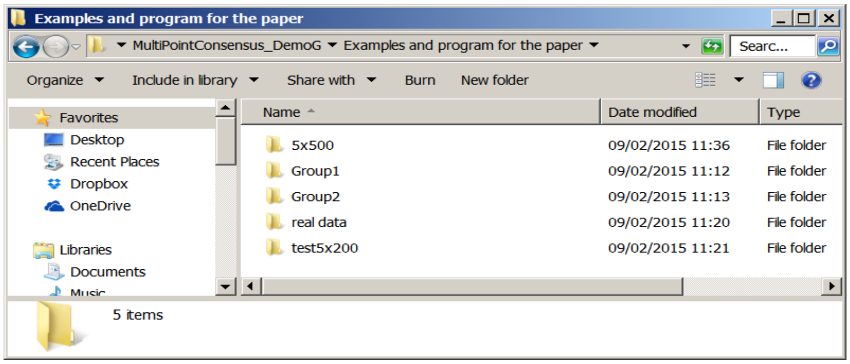

Thus, for example Ex1-2 of Group 2, **Run** the program as shown below and then click “Start” button.

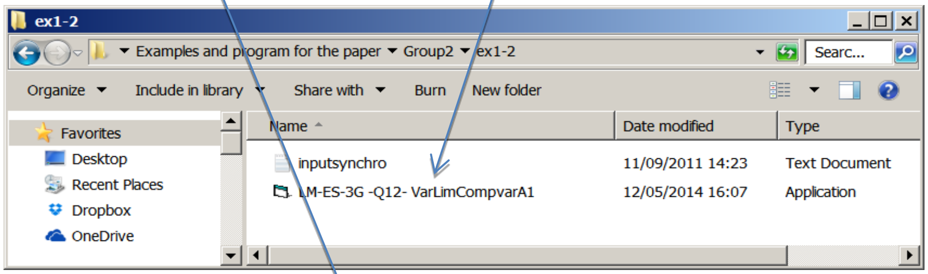

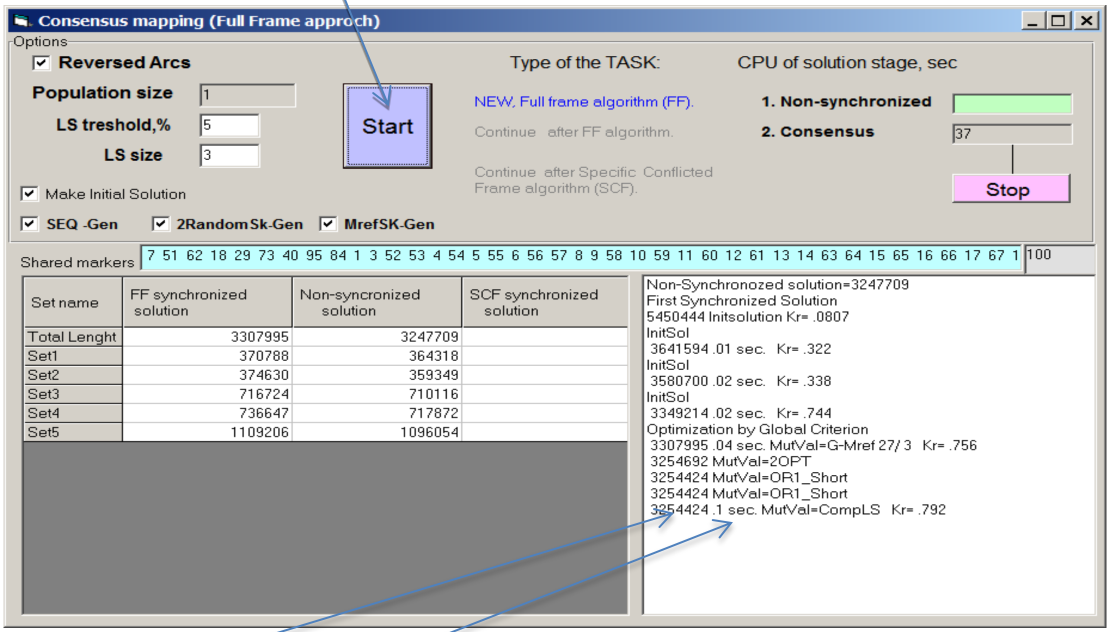

The “Total length“ and the CPU time values of the example will be represented in the output frame.

After starting, the program first creates non-synchronized solutions for each of the single data sets (Set1…Set5). Then, based on these single-map solutions, the program searches the consensus map. Note that the non-synchronized solution step is not taken into account in calculating the CPU time in the output frame. In this example, CPU time for the consensus solution was 0.1 sec (on our PC). Since usually evolution strategy algorithms do not finish the optimization process automatically based on the quality of the current achieved solution, the user has to stops the solution process by pressing the "STOP" button. After stopping the program, the "outputsynchro.txt" file is written in the same subfolder.

4. The structure of the input file.

Each dataset of the input data contains information about matrix distance for each single map, list and number of markers of the single map, and alias names of the markers (code of the shared markers accepted for the distance matrix). The structure of the matrix distance (based on pairwise recombination rates within each dataset) is a follows:

i ***j*** ***d***(i, j),

where d is distance (in recombination rate × 10000) between markers i and j. Other information in the input data is shown below using the example Ex1-2 of Group 2.

0 ← **code type of the solver** (a technical parameter not used in this version).

5 ← **number of the data sets** (single maps).

100 ← **number of shared markers** (across the entire dataset).

← **alias names of the markers**.

10 100 12 14 16 18 2 20 22 24 26 28 30 32 34 36 38 4 40 42 44 46 48 50 52 54 56 58 6 60 62 64 66 68 70 72 74 76 78 8 80 82 84 86 88 90 92 94 96 98 1 11 13 15 17 19 21 23 25 27 29 3 31 33 35 37 39 41 43 45 47 49 5 51 53 55 57 59 61 63 65 67 69 7 71 73 75 77 79 81 83 85 87 89 9 91 93 95 97 99

Zero ← **data divider** (a technical parameter sub-diving the data into data sets).

50 ← **number of markers of Set1.**

list of markers in Set1.

1 2 3 4 5 6 7 8 9 10 11 12 13 14 15 16 17 18 19 20 21 22 23 24 25 26 27 28 29 30 31 32 33 34 35 36 37 38 39 40 41 42 43 44 45 46 47 48 49 50

100 **size of matrix distance**

1 1 0 **distance d(1, 1) = 0**

1 2 41598 **distance d(1, 2) = 0.41598 × 10000).**

.

.

i j d **for other marker pairs, matrix distance of the Set1.**

.

.

Zero **data divider**

Same format for data of Sets 2-5.

5. The structure of the output file

The number of sets (single maps)

Total length of the consensus map

5 3254424

The number of markers of Set1

Total length of the single map1

50 364318

The marker order of the single map1

2 4 6 8 10 12 14 16 18 20 22 24 26 28 30 32 34 36 38 40 42 44 46 48 50 52 54 56 58 60 62 64 66 68 70 72 74 76 78 80 82 84 86 88 90 92 94 96 98 100

50 359349
1 3 5 7 9 11 13 15 17 19 21 23 25 27 29 31 33 35 37 39 41 43 45 47 49 51 53 55 57 59 61 63 65 67 69 71 73 75 77 79 81 83 85 87 89 91 93 95 97 99

50 714427
2 4 6 8 10 12 14 16 18 20 22 24 26 28 30 32 34 36 38 40 42 44 46 48 50 52 54 56 58 60 62 64 66 68 70 72 74 76 78 80 82 84 86 88 90 92 94 96 98 100

50 719704
1 3 5 7 9 11 13 15 17 19 21 23 25 27 29 31 33 35 37 39 41 43 45 47 49 51 53 55 57 59 61 63 65 67 69 71 73 75 77 79 81 83 85 87 89 91 93 95 97 99

100 1096626
2 1 3 4 6 5 7 9 8 10 12 11 13 14 15 16 17 18 19 21 20 22 23 24 25 26 27 28 29 30 32 31 33 34 35 36 37 38 39 40 42 41 43 44 45 46 47 48 49 51 50 52 53 55 54 56 57 58 59 61 60 62 64 63 65 66 67 68 69 70 71 72 73 74 75 76 77 78 79 80 81 82 83 84 85 86 87 88 90 89 92 91 93 94 95 96 97 98 100 99

100 ← **number of shared markers**

Consensus order:
2 1 3 4 6 5 7 9 8 10 12 11 13 14 15 16 17 18 19 21 20 22 23 24 25 26 27 28 29 30 32 31 33 34 35 36 37 38 39 40 42 41 43 44 45 46 47 48 49 51 50 52 53 55 54 56 57 58 59 61 60 62 64 63 65 66 67 68 69 70 71 72 73 74 75 76 77 78 79 80 81 82 83 84 85 86 87 88 90 89 92 91 93 94 95 96 97 98 100 99

The Total length of the consensus map

LSbestSK= 3254424

The coefficient of recovery

Kr=0 .792

The CPU time after stopping the program (note, it is not CPU for solving the test problem).

6.61 sec.
